# Low Synovial Lymphocyte Percentage as a Novel Diagnostic Marker of Periprosthetic Joint Infection

**DOI:** 10.2106/JBJS.OA.25.00241

**Published:** 2025-11-03

**Authors:** Assil-Ramin Alimy, Jonah Sijakov, Nico Maximillian Jandl, Patricia Bartsch, Christian Ries, Jan Hubert, Eva Tolosa, Holger Rohde, Johannes Keller, Frank Timo Beil, Tim Rolvien

**Affiliations:** 1 Department of Trauma and Orthopedic Surgery, University Medical Center Hamburg-Eppendorf, Hamburg, Germany; 2 Institute of Medical Microbiology, Virology and Hygiene, University Medical Center Hamburg-Eppendorf, Hamburg, Germany; 3 Helios ENDO-Klinik Hamburg, Hamburg, Germany; 4 Institute of Immunology, University Medical Center Hamburg-Eppendorf, Hamburg, Germany

## Abstract

**Objectives:**

Correctly diagnosing periprosthetic joint infection (PJI) remains a major clinical challenge. While acute PJI is straightforward to identify because of its pronounced clinical presentation, chronic infections remain challenging to detect since clinical signs are subtle and standard criteria lack sensitivity. By analyzing a wide range of serum and synovial parameters in patients undergoing revision arthroplasty, we sought to identify the most accurate diagnostic PJI markers.

**Methods:**

A retrospective analysis of 400 patients undergoing knee or hip revision arthroplasty, including 145 patients who had PJI and 255 patients who had aseptic failure, was conducted. Diagnosis of PJI was established using the 2018 International Consensus Meeting criteria. A comprehensive evaluation of medical records, serum, and synovial biomarkers was performed. For each marker, receiver operating characteristic curves, calculating the area under the curve and optimal cutoff values, were determined.

**Results:**

Synovial biomarkers such as alpha-defensin and C-reactive protein did not demonstrate superior diagnostic performance compared with polymorphonuclear neutrophil (PMN) count and percentage. Importantly, low synovial lymphocyte percentage (<34.7%) emerged as the most accurate marker for PJI diagnosis (area under the receiver operating characteristic curve [AUC] = 0.96, sensitivity = 0.85, specificity = 0.96), independent of location or infection subtype (acute and chronic). This was further supported by a conditional inference tree model for diagnosing chronic PJI, which identified synovial lymphocyte percentage and PMN count as key decision nodes and demonstrated excellent diagnostic performance (AUC = 0.95; sensitivity = 0.93; specificity = 0.91).

**Conclusions:**

Our study provides evidence that low synovial lymphocyte percentage is a reliable diagnostic marker of PJI. Integrating the assessment of synovial lymphocytes into clinical practice could enable more timely diagnosis and, therefore, effective treatment, ultimately improving patient outcomes. However, as this was a single-center study limited to hip and knee revision, external validation is warranted to confirm the generalizability of our findings.

**Level of Evidence:**

Prognostic Level III. See Instructions for Authors for a complete description of levels of evidence.

## Introduction

Periprosthetic joint infection (PJI) remains one of the most challenging complications following total hip and knee arthroplasty^1-3^. With projections indicating a continued increase in arthroplasty procedures, the clinical burden of PJI is expected to rise accordingly^4^.

For effective treatment, accurate diagnosis of PJI is essential. Over the past few decades, multiple consensus frameworks have defined PJI by combining clinical findings with preoperative serum and synovial laboratory markers, as well as preoperative and intraoperative microbiological analysis^5-9^. However, diagnosing PJI remains challenging, especially in chronic and low-grade infections^10^. No single test or biomarker has demonstrated optimal accuracy, and considerable uncertainty remains regarding which parameter is best suited for reliably diagnosing PJI^11,12^. Recent advances in molecular diagnostics, such as metagenomic next-generation sequencing, have shown promise in improving pathogen detection, especially in culture-negative infections^13,14^. While these technologies represent an important future direction in the diagnostic workup of chronic PJI, they are not yet universally available and face issues of cost and standardization^15^. Furthermore, synovial alpha-defensin, as a relatively novel biomarker, has not consistently demonstrated diagnostic superiority over established synovial parameters, particularly in chronic or low-grade infections^16-18^. Thus, there remains a need to enhance preoperative diagnostic accuracy to inform clinical decision-making better.

In this study, we conducted an in-depth analysis of a broad range of serum and synovial parameters in a cohort of patients undergoing knee or hip revision arthroplasty to identify more accurate and reliable markers for correct PJI diagnosis. We aim to provide clinicians with practical guidance and diagnostic tools that are both accessible and readily implementable in routine clinical practice for diagnosing PJI.

## Methods

In brief, this retrospective study reviewed 400 patients undergoing revision hip or knee arthroplasty between 2015 and 2024, applying the International Consensus Meeting (ICM) 2018 criteria to diagnose PJI^10^. A total of 145 patients met diagnostic criteria, with infections classified as acute (n = 44), chronic (n = 79), or sinus tract PJI (n = 22) (Table I, Supplemental Tables 1 and 2). Acute PJI was mainly caused by *Staphylococcus aureus* and *Streptococcus dysgalactiae*, while chronic PJI was associated with low-virulence organisms (Supplemental Table 3). Patients who did not fulfil the ICM criteria were categorized as aseptic and underwent revision surgery for a range of conditions (Supplemental Table 4). Laboratory analyses included serum and synovial fluid cytology and synovial alpha-defensin. Advanced statistical methods, including principal component analysis (PCA), regression, and receiver operating characteristic (ROC) analysis, were used to evaluate the diagnostic accuracy of preoperatively available serum and synovial biomarkers in relation to PJI diagnosis in the presence of the complete set of ICM 2018 criteria, including the results of histological and microbiological analysis from tissue samples. A detailed description of the methods is presented in the Supplementary Methods.

**TABLE I:** Overview of the Study Cohort Stratified About the Presence of PJI

	Overall (n = 400)	PJI (n = 145)	Aseptic Revision (n = 255)	p
Demographics				
Age in years (mean, SD)	70.6 (11.7)	71.2 (11.9)	70.3 (11.6)	0.463
Body mass index (mean, SD)	29.6 (6.6)	29.1 (7.3)	29.9 (6.2)	0.269
Men (n, %)	181 (45.2)	87 (60.0)	94 (36.9)	**<0.001**
Location				
Revision THA (n, %)	206 (51.5)	82 (56.6)	124 (48.6)	0.127
Revision TKA (n, %)	194 (48.5)	63 (43.5)	131 (51.4)	
Most common bacteria hip (n, %)/knee (n, %)				
CoNS		18 (22.0)/6 (9.5)	NA	
*Staphylococcus aureus*		13 (15.9)/13 (20.6)	NA	
*Escherichia coli*		4 (4.9)/2 (3.2)	NA	
Polymicrobial		11 (13.4)/5 (7.9)	NA	
Most frequent indications for aseptic revision surgery of the hip (n, %)				
Loosening		NA	40 (32.3)	
Recurrent dislocation		NA	36 (29.0)	
Periprosthetic fracture		NA	16 (12.9)	
Most common indications for aseptic revision surgery of the knee (n, %)				
Instability		NA	29 (22.1)	
Loosening		NA	25 (19.1)	
Retropatellar osteoarthritis		NA	21 (16.0)	

CoNS = coagulase-negative Staphylococci, THA = total hip arthroplasty, and TKA = total knee arthroplasty.

Bold indicates significant differences (P < 0.05).

## Results

### ICM 2018 Criteria Allow Broad Differentiation of Patients with PJI

**Fig. 1:**
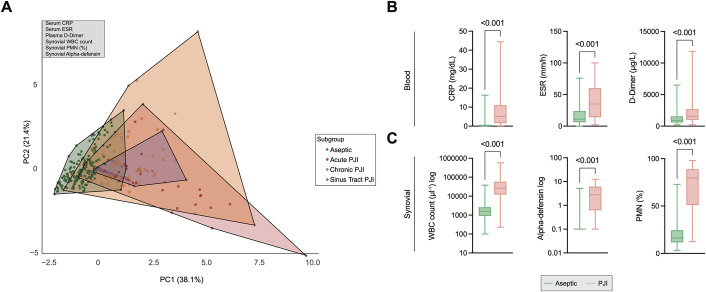
Blood and synovial parameters according to current diagnostic criteria enable broad differentiation between patients with PJI and those undergoing aseptic revision. **Fig. 1-A** Visualization of patients with different subtypes of PJI and patients undergoing aseptic revision surgery using PCA based on serum and synovial parameters according to ICM 2018 criteria. Comparison of (**Fig. 1-B**) serum and (**Fig. 1-C**) synovial fluid biomarkers between aseptic revision and PJI cases. ESR = erythrocyte sedimentation rate, ICM = International Consensus Meeting, PCA = principal component analysis, PJI = periprosthetic joint infection, PMN = polymorphonuclear neutrophils, and WBC = white blood cell count.

PCA differentiated PJI from aseptic cases (Fig. 1-A). However, several patients, particularly those with chronic PJI, clustered closely with aseptic cases (silhouette score = 0.12). Comparisons of blood and synovial biomarkers further revealed higher serum C-reactive protein (CRP), erythrocyte sedimentation rate, and D-dimer in PJI, along with higher synovial white blood cell (WBC) count, polymorphonuclear neutrophil (PMN) percentage, and alpha-defensin (Figs. 1-B and 1-C).

### Serum WBC Differential Demonstrates Limited Utility in Chronic PJI

**Fig. 2:**
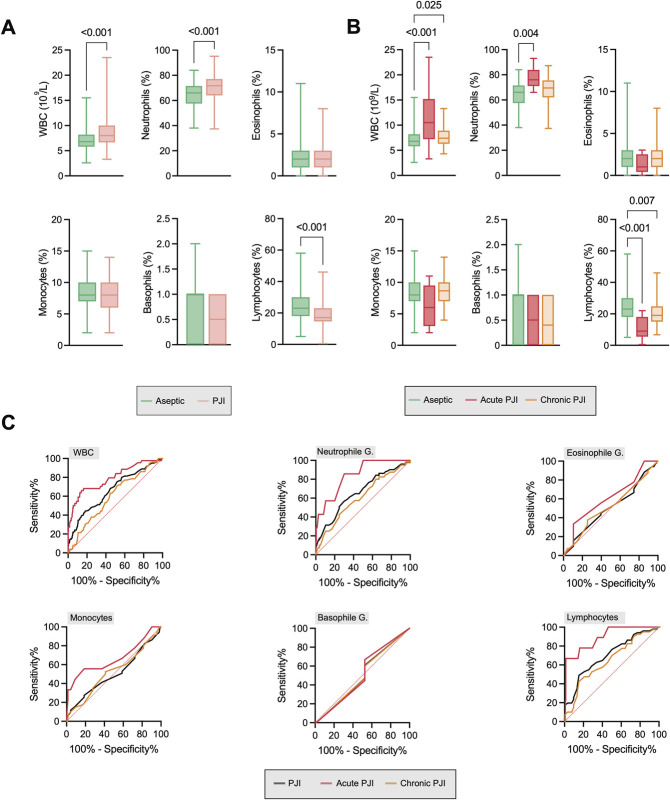
Serum white blood cell differential analysis has limited value in diagnosing patients with chronic PJI. **Fig. 2-A** Quantification of WBC count and the relative fractions of neutrophils, eosinophils, monocytes, basophils, and lymphocytes in patients with aseptic failure and PJI. **Fig. 2-B** Comparison of the same parameters across subgroups of acute and chronic PJI compared with aseptic cases. **Fig. 2-C** ROC curve analyses for WBC count, neutrophils, eosinophils, monocytes, basophils, and lymphocytes. PJI = periprosthetic joint infection, ROC = receiver operating characteristic, and WBC = white blood cell.

We extended our analysis to include serum parameters, specifically WBC differential, where WBC count, neutrophils (%), and lymphocytes (%) demonstrated differences (Fig. 2-A). WBC counts were elevated in both acute and chronic PJI (Fig. 2-B). ROC curve analyses showed modest diagnostic accuracy for WBC count, neutrophils (%), and lymphocytes (%), particularly in acute but to a lesser extent in chronic PJI (Fig. 2-C).

### Low Synovial Lymphocyte Percentage as a Key Feature of Immune Cell Composition Shifts During PJI

**Fig. 3:**
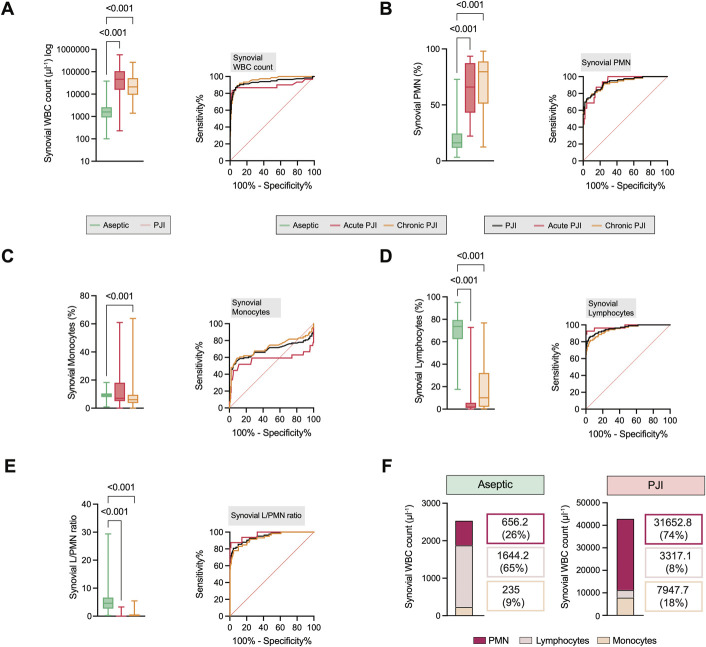
Synovial WBC differential demonstrates a shift toward reduced synovial lymphocyte percentage in PJI. Quantification and comparison between aseptic revision and PJI (including differentiation between acute and chronic) and diagnostic accuracy (ROC curve) for each parameter to correctly diagnose PJI, for synovial (**Fig. 3-A**) WBC count, (**Fig. 3-B**) PMN percentage, (**Fig. 3-C**) monocyte percentage, (**Fig. 3-D**) lymphocyte percentage, and (**Fig. 3-E**) lymphocyte-to-PMN ratio. **Fig. 3-F** Distribution of the average (mean) synovial immune cell composition in patients with aseptic failure and patients with PJI. PJI = periprosthetic joint infection, PMN = polymorphonuclear neutrophils, ROC = receiver operating characteristic, and WBC = white blood cell.

Synovial WBC count, alpha-defensin, PMN percentage, and absolute PMN count demonstrated discriminatory performance, with high area under the receiver operating characteristic curve (AUC) values in both acute and chronic PJI (Figs. 3-A and 3-B, Supplemental Figs. 1-A and 1-B). Synovial CRP was elevated in acute and chronic PJI (Supplemental Fig. 1-C). Absolute monocyte count was increased in PJI overall, and the proportion (median) of monocytes was reduced only in chronic PJI (Fig. 3-C, Supplemental Fig. 1-D). Interestingly, synovial lymphocytes emerged as a valuable diagnostic marker. Although the absolute number of synovial lymphocytes increased during PJI, the proportion was reduced in both acute and chronic PJI (Fig. 3-D, Supplemental Fig. 1-E). Synovial lymphocyte percentage further allowed moderate differentiation between acute and chronic PJI (cutoff = 5.8%, AUC = 0.70, 95% confidence interval [CI]: 0.59-0.82, sensitivity = 0.65, specificity = 0.77). To explore whether combining immune cell populations could enhance diagnostic accuracy, we additionally assessed ratios such as the lymphocyte-to-PMN and lymphocyte-to-monocyte ratios. However, these derived metrics did not improve performance beyond synovial lymphocyte percentage alone (synovial lymphocyte-to-PMN ratio, acute: p = 0.502; chronic: p = 0.513, lymphocyte-monocyte to PMN ratio, acute: p = 0.213; chronic: p = 0.258) (Fig. 3-E, Supplemental Figs. 2-A, 2-B and 2-C). Furthermore, to evaluate whether combining synovial immune cell populations improves diagnostic accuracy, we performed a logistic regression model incorporating both lymphocyte percentage and PMN count. The combination achieved an AUC of 0.96 (95% CI: 0.93-0.99), with sensitivity of 0.85 and specificity of 0.96. However, this performance was not significantly different from lymphocytes alone (p = 0.730), indicating that synovial lymphocyte percentage alone provides a strong discriminatory value in chronic PJI. To better understand changes in cellular composition, we analyzed the average (mean) absolute counts of PMN, lymphocytes, and monocytes in synovial fluid (Fig. 3-F). We observed high frequencies across cell populations in PJI, with particularly striking elevations in PMNs and monocytes (Fig. 3-F).

### Proportion of Synovial Lymphocytes as a Standalone Key Diagnostic Marker for Patients with PJI

Effect size analysis comparing PJI and aseptic cases identified synovial lymphocyte percentage as the most discriminative marker, which was supported by diagnostic-accuracy comparisons (Supplemental Figs. 3-A and 3-B, Supplemental Table 5). Stratified by joint, the top 3 markers by effect size in hip PJI matched the overall cohort, and rankings were similar in knee PJI (Supplemental Figs. 3-C through 3-F, Supplemental Tables 6 and 7). In acute PJI, synovial lymphocyte percentage showed the largest effect size (Supplemental Figs. 4-A and 4-B, Supplemental Table 8). Sex-specific analyses revealed no significant differences in key synovial parameters across various subgroups (Supplemental Figs. 5 and 6).

**Fig. 4:**
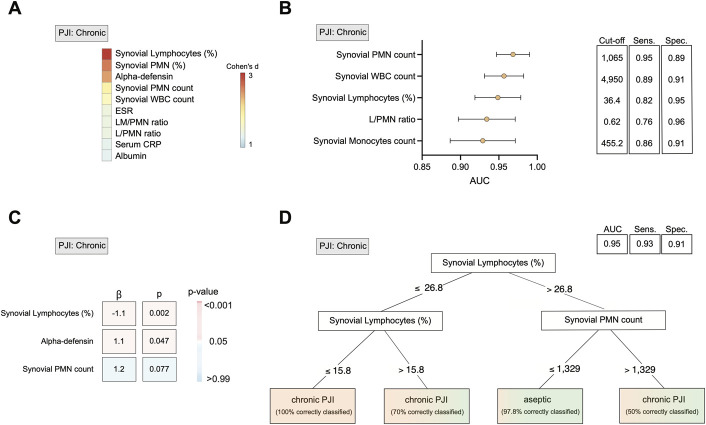
Ranking of the most reliable diagnostic parameters and clinical decision aid confirm the value of synovial lymphocyte percentage for diagnosing chronic PJI. **Fig. 4-A** Quantification of effect sizes (Cohen d) for various parameters comparing patients with aseptic failure and chronic PJI. Color intensity of Cohen d values reflects the magnitude of the effect size. **Fig. 4-B** Forest plot displaying AUC and diagnostic accuracy for identifying chronic PJI. **Fig. 4-C** Heatmap derived from multiple logistic regression models evaluating the predictive value of synovial lymphocyte percentage, alpha-defensin, and synovial PMN count for chronic PJI. The standardized regression coefficient (β) is displayed for each variable. Color coding reflects the p-value. **Fig. 4-D** Conditional inference tree for diagnosing chronic PJI using synovial PMN count, synovial WBC count, and synovial lymphocyte percentage as predictors. Color coding of the classification tree corresponds to the classification of patients. AUC = area under the receiver operating characteristic curve, PJI = periprosthetic joint infection, PMN = polymorphonuclear neutrophils, L/PMN = lymphocyte-to-PMN ratio, LM/PMN = lymphocyte/monocyte-to-PMN ratio, and WBC = white blood cell.

In chronic PJI, the synovial lymphocyte percentage again emerged as the most distinctive marker, demonstrating the highest effect size (Fig. 4-A). In terms of diagnostic performance, synovial lymphocyte percentage also ranked high (Fig. 4-B, Supplemental Table 9). Given the strong performance of synovial lymphocytes, alpha-defensin, and synovial PMN count, we evaluated them as independent predictors of chronic PJI using logistic regression analysis (Fig. 4-C). The full model showed strong explanatory power, with Nagelkerke R^2^ = 0.648 and Cox and Snell R^2^ = 0.389. Goodness-of-fit was assessed using several model comparison metrics. Compared with the intercept-only model (M_0_), the full logistic regression model (M_1_) demonstrated a significant improvement in fit, as indicated by a large reduction in deviance (Δχ^2^ = 97.64, df = 3, p < 0.001), a decrease in an Akaike information criterion (from 183.61 to 91.96), and a lower Bayesian information criterion (BIC) (from 186.89 to 105.11). A conditional inference tree (CIT) analysis was further performed to support clinical decision-making in the diagnosis of chronic PJI (Fig. 4-D). Overall, the CIT model selected synovial lymphocyte percentage and absolute PMN count as key decision nodes and demonstrated excellent classification performance (AUC = 0.95, 95% CI: 0.91-0.99). To evaluate generalizability and address potential overfitting, we performed internal validation using 10-fold cross-validation (AUC = 0.91, 95% CI: 0.85-0.96) and bootstrap resampling (1,000 iterations; AUC = 0.96, 95% CI: 0.90-0.97) (Fig. 4-D).

## Discussion

PJI remains one of the most serious and impactful complications following arthroplasty^19,20^. Despite recent advances, diagnosis remains challenging, especially for chronic, low-virulence cases with subtle findings. However, given that therapeutic decisions critically depend on an accurate diagnosis, optimizing the diagnostic workup of PJI remains a key challenge. In this study, we provide evidence that synovial lymphocyte percentage is a valuable, simple, and underutilized biomarker for correctly diagnosing PJI.

Routine serum WBC differential analysis did not provide additional value, particularly in the context of chronic PJI. This underlines the findings of previous studies that serum WBC differential does not provide additional benefit in diagnosing PJI^21,22^. While markers such as total WBC count, PMN percentage, and lymphocyte percentage showed reasonable diagnostic accuracy in acute PJI, their utility was markedly diminished in chronic infections. Therefore, serum WBC differential, although potentially useful in acute cases, should not be relied on as a standalone tool in diagnostically ambiguous scenarios, particularly in chronic PJI where more specific synovial markers are needed. This is further underscored by the superior performance of synovial metrics, which more accurately reflect the localized immune response within the joint space.

For synovial analysis, low percentage of synovial lymphocytes emerged as a valuable diagnostic parameter for PJI, independent of joint location and onset. It may be used for further consideration in combination with absolute PMN count, particularly for chronic PJI. Although the absolute count of synovial lymphocytes in PJI is, on average, about twice as high as in aseptic revision cases, the relative reduction in lymphocyte percentage seems to result from a more pronounced expansion of other immune cell populations, particularly PMN and monocytes, rather than from suppressed lymphocyte proliferation. While the relative decrease of synovial lymphocytes in PJI may not have direct pathomechanistic implications, the combined deviation by both PMN and monocytes appears to result in low synovial lymphocyte percentage being the most appropriate marker to determine whether a patient has PJI. This interpretation is supported by our finding that the lymphocyte-to-PMN ratio adds no diagnostic value beyond synovial lymphocyte percentage, consistent with prior reports that the neutrophil-to-lymphocyte ratio is not superior to relative PMN percentage^23^.

Generally, research on lymphocyte populations in the synovial fluid remains scarce. One study assessed multiple synovial biomarkers and observed a marked reduction in lymphocytes among PJI patients, with an average value of 5.4%^24^. Nonetheless, neutrophil and monocyte fractions, as well as the absolute synovial WBC count, were found to outperform lymphocytes in diagnostic performance^24^. The discrepancy between those findings and our results may be explained by differences in study populations. The study did not perform subgroup analyses based on timing of patients with PJI and likely included a predominance of acute cases, as well as a patient population in which 90% of infections involved the knee joint. In our cohort, while synovial lymphocytes emerged as a valuable marker, we similarly found that the absolute PMN count remained superior regarding overall diagnostic performance for PJI of the knee. Given its robust performance in differentiating PJI from aseptic failure, synovial lymphocyte percentage may serve as a valuable adjunct to established synovial markers such as leukocyte esterase, WBC count, and PMNs. While these findings are promising, further validation in larger multicenter cohorts is needed before its inclusion in formal diagnostic criteria can be recommended.

Our study has several limitations. One limitation of our study is the restriction to one set of diagnostic criteria. Although multiple PJI definitions exist and no universal standard is endorsed, we used the ICM criteria because they are routinely applied at our institution and offer a balanced, pragmatic approach^25,26^. Another limitation is that we excluded patients with sinus tract-associated PJI from our diagnostic analyses. While this may limit the generalizability of our findings to all PJI presentations, we intentionally focused on cases that were diagnostically uncertain. Sinus tracts represent a major diagnostic criterion in the ICM definition and typically allow immediate clinical diagnosis without the need for supporting laboratory markers. Therefore, including these cases would likely have inflated diagnostic performance metrics and detracted from the study's primary objective of identifying reliable markers for diagnostically challenging PJI cases. Another limitation is that we did not include novel, synovial biomarkers that may further reflect the local inflammatory response, such as interleukin-6 (IL-6). Therefore, future studies should further evaluate synovial lymphocyte percentage in combination with established and emerging biomarkers, such as IL-6, to determine whether multimarker panels can further enhance diagnostic accuracy in PJI. Another limitation of our study is the lack of external validation to confirm our findings regarding synovial lymphocyte percentage. Therefore, we encourage further studies to challenge and refine our findings on the importance of considering synovial lymphocyte percentage. However, we did perform internal validation, which underlined our exploratory analysis and confirmed the major role of synovial lymphocytes. Although synovial cytology was performed using standardized protocols within a single certified laboratory, potential intraobserver variability and limited generalizability across laboratories may influence lymphocyte percentage measurements and should be considered in future multicenter validation studies. Moreover, our study focused solely on hip and knee revision arthroplasties. Thus, the diagnostic performance of synovial lymphocyte percentage in other joints, such as the shoulder or elbow, remains to be determined.

In conclusion, our study provides evidence that low synovial lymphocyte percentage is a reliable and practical indicator of PJI. We further highlight the importance of evaluating immune cell composition, especially relative WBC proportions, rather than relying solely on absolute counts, to gain a comprehensive understanding of the adaptations in PJI. Thus, establishing these parameters may ultimately improve the diagnosis of PJI, thereby facilitating timely, effective treatment.

## Appendix

Supporting material provided by the authors is posted with the online version of this article as a data supplement at jbjs.org (http://links.lww.com/JBJSOA/A991). This content was not copyedited or verified by JBJS.
